# Cessation of breastfeeding and associated factors in the era of elimination of mother to child transmission of HIV at Ndejje health center, Uganda: a retrospective cohort study

**DOI:** 10.1186/s13006-020-00323-7

**Published:** 2020-09-07

**Authors:** Jackslina Gaaniri Ngbapai, Jonathan Izudi, Stephen Okoboi

**Affiliations:** 1 Institute of Public Health and Management, Clarke International University, P.O. Box 7782, Kampala, Uganda; 2 Department of Community Health, Faculty of Medicine, Mbarara University of Science and Technology, P.O Box 1410, Mbarara, Uganda; 3 Infectious Diseases Institute, Makerere University College of Health Sciences, P.O. Box 22418, Kampala, Uganda; 4 Global Health Institute, University of Antwerp, Antwerp, Belgium

**Keywords:** Breastfeeding, HIV exposed infant, Mother to child transmission of HIV, And option B plus

## Abstract

**Background:**

Breastfeeding an infant exposed to Human Immunodeficiency Virus (HIV) carries the risk of HIV acquisition whilst not breastfeeding poses a higher risk of death from malnutrition, diarrhea, and pneumonia. In Uganda, mothers living with HIV are encouraged to discontinue breastfeeding at 12 months but data are limited. We examined the frequency and factors associated with cessation of breastfeeding at 1 year among mothers living with HIV at Ndejje Health Center IV, a large peri-urban health facility in Uganda.

**Methods:**

This retrospective cohort study involved all mothers living with HIV and enrolled in HIV care for ≥12 months between June 2014 and June 2018. We abstracted data from registers, held focus group discussions with mothers living with HIV and key informant interviews with healthcare providers. Cessation of breastfeeding was defined as the proportion of mothers living with HIV who had discontinued breastfeeding at 1 year. We summarized quantitative data descriptively, tested differences in outcome using Chi-square and *t* - tests, and established independently associated factors using modified Poisson regression analysis at 5% statistical significance level. We thematically analyzed qualitative data to enrich and triangulate the quantitative results.

**Results:**

Of 235 participants, 150 (63.8%) had ceased breastfeeding at 1 year and this was independently associated with the infant being male (Adjusted Risk Ratio [aRR] 1.25, 95% confidence interval [CI] 1.04, 1.50), the mother being multiparous (aRR 1.26, 95% CI 1.04–1.53), and the initiation of breastfeeding being on the same-day as birth (aRR 0.06, 95% CI 0.01–0.41). The reasons for ceasing breastfeeding included male infants over breastfeed than females, maternal literacy and knowledge adequacy about breastfeeding, support and reminders from the partner, and boys can bite once they get teeth.

**Conclusion:**

Suboptimal proportion of infants were ceased from breastfeeding at 1 year and this might increase the risk of mother to child transmission of HIV. Cessation of breastfeeding was more likely among male infants and multiparous mothers but less likely when breastfeeding was initiated on the same-day as birth. Interventions to enhance cessation of breastfeeding should target none multiparous mothers and those with female infants.

## Background

In sub-Sahara African region (SSA), an estimated 60% of infants born to mothers living with Human Immunodeficiency Virus (HIV) acquire HIV during pregnancy, delivery, or breastfeeding [1]. In Uganda, the rate of mother to child transmission of HIV (MTCT) falls within the range of 5–30% [1], and the Joint United Nations Programme on HIV/AIDS estimates that 1.4 million people were living with HIV, with 740,000 being women aged ≥15 years in 2018 [2]. Following the scale up of elimination of mother to child transmission of HIV (EMTCT) Option B Plus policy, at least 90% of pregnant women living with HIV are now on lifelong anti-retroviral therapy (ART) [3]. The increasing number of women on EMTCT program is attributed to accreditation of many lower level health facilities to provide ART to both pregnant and breastfeeding women living with HIV [4]. Although EMTCT has led to an estimated 86% reduction in HIV infection among children between 2010 and 2016, 14% of infants are infected with HIV during breastfeeding, which is still high [5].

In mothers living with HIV, breastfeeding is recommended because it protects infants exposed to HIV (hereafter termed as infants) from morbidity and mortality. A scoping review of breastfeeding among pregnant women living with HIV included 35 studies in sub Saharan Africa, found that healthcare workers’ personal biases, inadequate counselling skills, mother’s limited knowledge about EMTCT, a culture of mixed feeding norms, maternal lack of decision-making power, limited follow-up of mothers after delivery, and HIV related stigma associated with replacement feeding are some of the factors that hinder cessation of breastfeeding at 1 year among women living with HIV [6].

Breastfeeding in the context of HIV has repercussions for infant and child survival [7, 8]. Therefore, balancing the risk of HIV acquisition through breastfeeding against the higher risk of death from malnutrition, diarrhea, and pneumonia by not breastfeeding presents a serious challenge [8]. Currently in Uganda, protecting the infant from the risk of death from these causes is considered as critical as circumventing HIV transmission through breastfeeding [8, 9]. The WHO recommends that mothers living with HIV breastfeed for at least 12 months and may continue breastfeeding for up to 24 months or longer thus similar to the general population while being fully supported for ART adherence [10]. However, the Uganda HIV prevention and treatment guidelines recommend that mothers living with HIV should be encouraged to discontinue (cease) breastfeeding at 12 months, provided the infant has tested negative for HIV at 12 months [7–9]. This enables mothers living with HIV to take the advantage of the maximum benefits of breastfeeding and to improve the chances of infant survival while reducing the risk of HIV transmission [7–9].

Ndejje Health Center IV is one of the several health facilities accredited in Wakiso district to provide Option B Plus, an EMTCT strategy. However, anecdotal observations at the EMTCT clinic indicates that some infants acquire HIV infection at 1 year due to prolonged breastfeeding. Second, data are limited on the magnitude and factors associated with cessation of breastfeeding at 1 year among mothers living with HIV. Our study therefore examined the frequency of cessation of breastfeeding at 1 year and the associated factors among mothers living with HIV at Ndejje Health Center IV, a large peri-urban health facility in Uganda. The findings of this study will inform the implementation of breastfeeding practices as per the recommendations of EMTCT guideline.

## Methods

### Study design and setting

We used qualitative and quantitative methods of data collection and both were implemented concurrently. For the quantitative component, we designed a retrospective cohort study using available data within the EMTCT program in which exposure to breastfeeding had occurred at some time in the past. For the qualitative component, we held interviews with healthcare providers as key informants and focus group discussions (FGDs) with mothers living with HIV to enrich and triangulate the quantitative results. The study design was largely quantitative and data convergence was established at results interpretation. The cohort consisted of all mothers living with HIV enrolled to the EMTCT program between June 2014 and June 2018 at Ndejje Health Center (HC) IV, a large peri-urban health facility in Makindye Division, Wakiso district, Uganda.

Ndejje HC IV is a county level health facility according to Uganda’s health system accreditation guidelines and it provides curative, preventive, rehabilitative, and promotive health services to an estimated 100,000 population [11]. Ndejje HC IV serves a substantial proportion of people living with HIV, largely from the eight divisions of Busabala, Masajja, Bunamwaya, Kigo, Mutungo, Ndejje, Sseguku, and Mutundwe. The health facility serves approximately 133,068 patients annually which is a high patient load compared to its threshold. Currently, the health facility has a total of 61 staff, nine of which are dedicated to the maternal and child health clinic.

The health facility implements the Uganda National Option B Plus policy [7–9], which we have fully described elsewhere [12, 13]. Under this policy, all pregnant mothers living with HIV are started on ART for life regardless of their immunological and clinical status and infants receive Nevirapine syrup as prophylaxis from birth until 6 weeks, adjusted according to weight and age bands.

At 6 weeks, Nevirapine prophylactic syrup is stopped and cotrimoxazole prophylaxis is introduced and a dry blood spot obtained for HIV test using Deoxyribonucleic Acid-Polymerase Chain Reaction (DNA-PCR). A second dry blood spot is obtained for DNA-PCR test at 9 months [9]. Infants receive exclusive breastfeeding for the first 6 months of life followed by complementary feeding. At 1 year, mothers are encouraged to discontinue breastfeeding and a third dry blood spot is obtained at exactly 6 weeks after cessation of breastfeeding for DNA-PCR test [8, 9]. A final diagnosis of HIV is performed at 18 months using a rapid antibody test. However, when infants seroconvert (test positive for HIV) at any of the testing time points, ART is started and cotrimoxazole prophylaxis is continued for life.

To minimize loss of mother-baby pairs and improve retention, Uganda introduced a concept of Mother-Baby Care Point (MBCP) which is a service delivery model in the maternal and child health clinic. The MBCP delivery model ensures mothers living with HIV and the infants are paired and cared for together until 18 months, a time when the final HIV infection status is determined [14]. Entry into the MBCP starts immediately after delivery because the mother and the infant have to receive HIV care as a single pair. The MBCP at Ndejje HC IV has four healthcare providers thus two midwives and two nursing officers, with one of them as the team leader. It operates 5 days a week, Monday to Friday, from 8.00 am to 5.00 pm, in accordance to the Uganda labor laws. The mothers routinely receive health education and counseling on various topics like prevention of mother to child transmission of HIV (PMTCT), nutrition, newborn care practices, maternal and newborn hygiene and breastfeeding among others.

Data are collected through paper-based system using HIV/ART care cards and early infant diagnosis of HIV (EID) and EMTCT registers, and later entered into an electronic database, the Open Medical Records System (Open-MRS). To ensure data quality, weekly data reviews are conducted alongside continuous quality improvement initiatives to address gaps in quality of HIV care.

Notable quality improvement initiatives at the MBCP have targeted improved recording and reporting of data in the EMTCT and EID registers, uptake of HIV testing at 6 weeks, and maternal nutritional assessment using Mid-upper arm circumference among others.

### Study population and sample size

The study population consisted of all mothers living with HIV enrolled to the EMTCT program for at least 1 year, and all of them were still receiving HIV care at the time of data abstraction. We excluded mothers living with HIV with infants below 1 year of age because cessation of breastfeeding is implemented at 1 year. So, it would be erroneous to measure cessation of breastfeeding in such mother-baby pairs. Also, mother-baby pairs transferred to other health facilities were excluded because it was not possible to obtain data on cessation of breastfeeding. We further excluded records for mother-baby pairs where the infant had died or seroconverted and those with gross missing data. We did not calculate a sample size but used census sampling as a retrospective cohort study that consisted of records review was used. Four FGDs of eight to 12 mothers living with HIV each were held, with the mothers randomly selected from amongst those attending the EMTCT clinic. Besides, four healthcare providers who were directly involved in providing care at the MBCP were purposively selected as key informants and interviewed.

### Study variables

Our outcome variable was cessation of breastfeeding at 1 year documented in the EID register, measured on a binary scale (yes or no). We defined cessation of breastfeeding as the proportion of mothers living with HIV who had discontinued to breastfeed an infant at 1 year.

The independent variables included: maternal variables such as age in years but later dichotomized as below 25 or 25 years and beyond, antenatal care attendance at last pregnancy measured as yes and no, number of antenatal care visits at last pregnancy, ART regimen categorized as Tenofovir (TDF) or Zidovudine (AZT) containing regimens, parity measured as primiparous, secundiparous, and multiparous, and place of delivery measured as health facility or home. The infant variables included age in months, sex (male or female), time of initiation of cotrimoxazole prophylaxis (before 6 weeks or 6 weeks and beyond), and initiation of breastfeeding on same-day as birth (yes or no). The topics for the FGDs included modes of mother to child transmission of HIV and the likely preventive measures, importance of breastfeeding, when to stop breastfeeding an infant, and reasons for stopping to breastfeed an infant before or after 1 year.

### Data collection

#### Qualitative data

To enrich and triangulate the quantitative results, we conducted qualitative interviews with mothers living with HIV and healthcare providers. In particular, we held four FGDs, each consisting of eight to 12 mothers living with HIV who were selected randomly from amongst those attending the EMTCT clinic. The group consisted of mothers who had ceased breastfeeding and those due for cessation of breastfeeding at 1 year. The FGDs were held within the premises of the health facility in the local language, “*Luganda”,* by two research assistants, JGP and MN, both female MPH postgraduate students trained in qualitative research methods. One research assistant (JGP) moderated all the FGDs while the other (MN) audio-recorded the responses and probed where necessary. Each FGD lasted for about 40–60 min on average.

The moderator encouraged all the group members to ask questions and to provide comments as much as possible, irrespective of the correctness.

Group dominance by some members of the FGD was minimized by directing some probing questions and comments to other members of the group who seemed to engage less in the discussions. For key informant interviews (KII), four healthcare providers namely, two Midwives and two nursing officers directly engaged in the provision of EMTCT services at the MBCP were purposively selected and interviewed to elicit their expert opinions on practices of cessation of breastfeeding among mothers living with HIV. The KII lasted for 30–45 min, also conducted within the health facility premises in English language, in a quiet and convenient room. Both FGDs and KIIs were held until saturation was reached [15].

#### Quantitative data

We reviewed the PMTCT and EID registers for all eligible participants and abstracted data using a standardized checklist between April and May 2019. Whenever data were missing in the EID and EMTCT registers, the HIV/ART care cards and the electronic database (Open MRS) were used to retrieve such data.

### Data processing and analysis

#### Qualitative data

We audio-recorded all interviews and transcribed them verbatim. To ensure accuracy in transcription, we correlated the audio-recordings with the transcripts by replaying it while reading through the transcripts. We exported the transcripts to NVivo, a qualitative data analysis software, for thematic analysis. Three reviewers JGP (MPH postgraduate student), JI (Public health specialist and mixed-methods research fellow), and SO (Public health specialist and research supervisor) read the transcripts thoroughly and independently, and identified emergent categories and themes through coding using inductive data analysis approach. Independent coding was used to prevent selective perception and interpretive biases in the coding process. JGP, JI and SO discussed the categories and themes in a group, and then developed explicit summaries describing each category and theme. Discrepancies in the emergent categories and themes were resolved by consensus and a final codebook was thereafter developed. The verbatim quotations were then used to enrich and triangulate the quantitative results.

#### Quantitative data

We single-entered quantitative data using Epi-Data version 3.1 [16], concurrent with quality control measures namely skip patterns, alerts, range and legal values, and then exported the data to Stata version 15 for analysis [17]. We analyzed numerical data using descriptive statistics of means and standard deviations, and categorical data using frequencies and percentages. The outcome variable was computed as the proportion of mothers living with HIV who had stopped breastfeeding an infant at 1 year.

We tested differences in proportions of cessation of breastfeeding at 1 year with categorical variables using the Chi-square test for large cell counts (five and more counts) and the Fisher’s exact test for smaller cell counts (less than five counts). To test mean differences in cessation of breastfeeding at 1 year with numerical variables such as age, we used the student’s *t* - test. We performed sensitivity analysis to examine the effect of missing data on cessation of breastfeeding feeding for two variables, infant sex and maternal ART regimen, at bivariate analysis.

We considered variables with probability values (*p* values) less than 5 % at bivariate analysis as statistically significant for univariable and multivariable analyses. Besides, variables such as maternal age, parity, and initiation of breastfeeding on same-day as birth were considered clinically relevant because they carried important clinical prognosis for cessation of breastfeeding at 1 year. Our data showed that the outcome variable was frequent (more than 10%). Accordingly, the odds ratio (OR) was not an appropriate measure of association because of overestimation [18, 19].

We hence used risk ratios (RR) for both unadjusted and adjusted analysis, computed using the modified Poisson regression analysis with robust error variance to control for mild violations of the assumptions of Poisson regression analysis. We reported each RR with subsequent 95% confidence intervals (CI). We noted that five (2.1%) observations were missing for the variable infant sex but did not impute them at multivariate analysis because they were fewer than 10%.

### Ethical issues

Clarke International University Research Ethics Committee, CIU-REC (reference #CIU-REC/0136), approved this study. We received administrative approval from the Health Department of Wakiso district (reference # CR: MSMC 220/1). Key informants and focus group participants provided a written informed consent after explaining the purpose and benefits of the research as well as the risks involved. Participation in the study was voluntary and participants were free to withdraw at any stage if they so wished. All participant data were handled with confidentiality and privacy, and individual identifiers were anonymized.

## Results

### Study profile

**Fig. 1:**
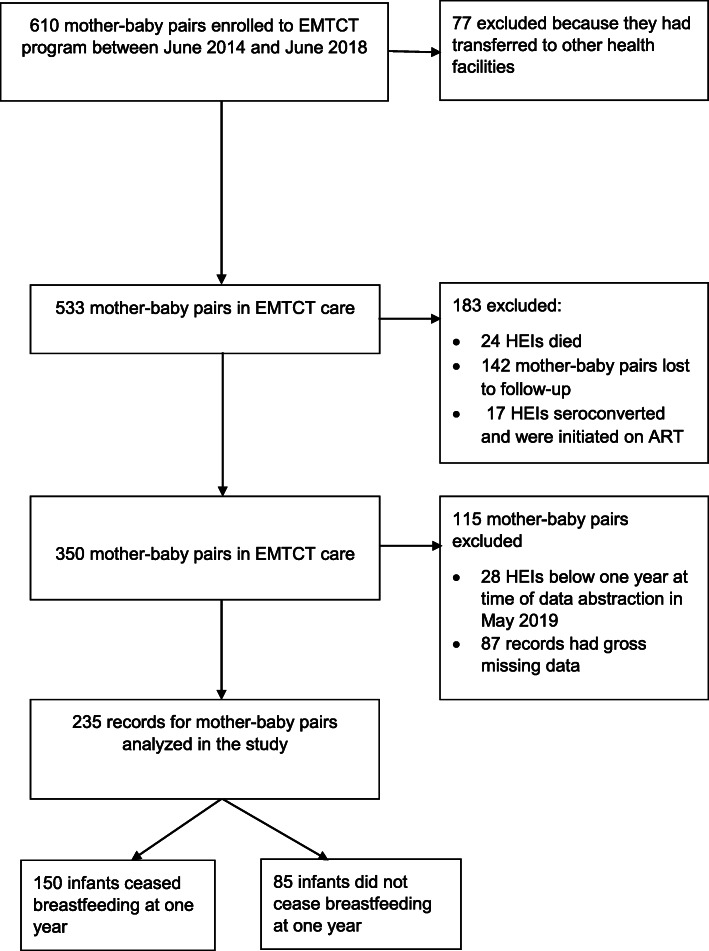
Study profile for mother-baby pairs enrolled to EMTCT at Ndejje HC IV between June 2014 and June 2018

Figure 1 presents a summary of the study profile. Our data shows that 610 mother-baby pairs were enrolled to the EMTCT program between June 2014 and June 2018. Of those enrolled, 77 were excluded because they had transferred to other health facilities. Of the remaining 533 mother-baby pairs, 183 were excluded for the following reasons: 24 infants had died, 142 mother-baby pairs were lost to follow-up, and 17 infants had seroconverted and were started on ART. Of 350 mother-baby pairs, another 115 were excluded: 28 infants were below 1 year of age and 87 had gross missing data. The final number of records analysed was for 235 mother-baby pairs.

### Sociodemographic characteristics of mothers living with HIV and their infants

Table 1 shows the sociodemographic characteristics of mother-baby pairs. Of 235 mothers living with HIV in the cohort, 138 (58.7%) were aged 16 to 25 years, 69 (29.4%) were multiparous, 119 (50.6%) had not attended antenatal care during the recent pregnancy, 220 (96.7%) had delivered in a health facility, and 116 (49.4%) gave birth to a male infant. The mean age of all the participants was 25.2 ± 4.8 years but ranged from 16 to 39 years. The median age was 24 years (IQR: 22–28 years).

**Table 1:** Sociodemographic characteristics of mothers living with HIV and the infants

Characteristics	Level	All (***n*** = 235) ***n*** (%)
Maternal age category	16–25	138 (58.7)
	25 and more	97 (41.3)
Age (continuous)	Mean Standard deviation	25.2 ± 4.8
Maternal parity	1	95 (40.4)
	2	71 (30.2)
	≥ 3	69 (29.4)
Attended antenatal care during recent pregnancy	Yes	96 (40.8)
	No	119 (50.6)
	Missing data	20 (8.5)
Place of delivery	Home	7 (3.1)
	Health facility	220 (96.9)
Infant sex	Female	114 (48.5)
	Male	116 (49.4)
	Missing data	5 (2.1)

### Bivariate analysis of cessation of breastfeeding at 1 year by maternal and infant factors

Table 2 summarized differences in cessation of breastfeeding at 1 year with respect to maternal and infant factors. Overall, our data showed that 150 (63.8%) mothers living with HIV had ceased breastfeeding at 1 year. A higher proportion of cessation of breastfeeding at 1 year was observed among mothers living with HIV who were in the age category of 16 to 25 years (54.0%), those who had not initiated breastfeeding on same-day as birth (98.7%), those who had not attended antenatal care during the recent pregnancy (54.0%), and those who had delivered in a health facility (96.6%). Similarly, a higher proportion was observed among those who had given birth to a male infant (56.0%) and among infants who had received cotrimoxazole prophylaxis before 6 weeks of age (52.7%).

**Table 2:** Bivariate analysis of cessation of breastfeeding at 1 year by maternal and infant factors

Characteristics	Ceased breastfeeding at 1 year		All (***n*** = 235)	***p*** value
	No (***n*** = 85)	Yes (***n*** = 150)		
Maternal age category	*n* (%)	*n* (%)	*n* (%)	0.051
16–25	57 (67.1)	81 (54.0)	138 (58.7)	
25 and more	28 (32.9)	69 (46.0)	97 (41.3)	
Mean ± Standard deviation	24.3 ± 0.52	25.7 ± 0.39	25.2 ± 4.8	0.027
Maternal parity				0.116
1	38 (44.7)	57 (38.0)	95 (40.4)	
2	29 (34.1)	42 (28.0)	71 (30.2)	
≥ 3	18 (21.1)	51 (34.0)	69 (29.4)	
Initiation of breastfeeding on same-day as birth				< 0.01
No	60 (70.6)	148 (98.7)	208 (88.5)	
Yes	25 (29.4)	2 (1.3)	27 (11.5)	
Attended antenatal care during recent pregnancy				0.020
Yes	34 (40.0)	62 (41.3)	96 (40.8)	
No	38 (44.7)	81 (54.0)	119 (50.6)	
Missing data	13 (15.3)	7 (4.7)	20 (8.5)	
Place of delivery				0.690
Home	2 (2.5)	5 (3.4)	7 (3.1)	
Health facility	79 (97.5)	141 (96.6)	220 (96.9)	
Time of cotrimoxazole prophylaxis initiation				0.040
< 6 weeks	28 (32.9)	79 (52.7)	107 (45.5)	
≥ 6 weeks	57 (67.1)	71 (47.3)	128 (54.5)	
Infant sex				0.002
Female	53 (62.3)	61 (40.7)	114 (48.5)	
Male	32 (37.6)	84 (56.0)	116 (49.4)	
Missing data	0 (0.0)	5 (3.3)	5 (2.1)	
Mothers ART regimen				0.08
AZT based regimen	7 (8.2)	3 (2.0)	10 (4.3)	
TDF based regimen	69 (81.2)	136 (90.7)	205 (87.2)	
Other regimens	6 (7.1)	6 (4.0)	12 (5.1)	
Missing data	3 (3.5)	5 (3.3)	8 (3.4)	

We observed statistically significant differences in cessation of breastfeeding with respect to initiation of breastfeeding on same-day as birth (*p* < 0.01), antenatal care attendance during recent pregnancy (*p* = 0.02), time of initiation of cotrimoxazole prophylaxis (*p* = 0.04) and infant sex (*p* = 0.002). Maternal age category demonstrated borderline statistical significance (*p* = 0.051). On average, the mothers who had ceased breastfeeding at 1 year were older than those who had not ceased breastfeeding: 25.7 ± 0.39 versus 24.3 ± 0.52 years respectively, (*p* = 0.027).

There was no statistically significant difference in cessation of breastfeeding with regard to parity, place of delivery, and ART regimen (all *p* > 0.05). Sensitivity analysis showed similar results of statistical significance for the variables infant sex and maternal ART regimen with cessation of breastfeeding in the presence and absence of missing data, suggesting that the results are robust to missing data.

### Factors associated with cessation of breastfeeding at 1 year

In unadjusted analysis (Table 3), our data showed that cessation of breastfeeding at 1 year was more likely when the infant was male than female (Unadjusted RR [URR] 1.35; 95% CI 1.10, 1.66), and when the mother was ≥25 years of age compared to those below 24 years of age (URR 1.21; 95% CI 1.00, 1.46). Conversely, cessation of breastfeeding was less likely when cotrimoxazole prophylaxis was initiated at or after 6 weeks of birth relative to initiation of cotrimoxazole before 6 weeks of birth (URR 0.87; 95% CI 0.79, 0.95), and when breastfeeding was initiated on same-day as birth compared to when it was on another day (URR 0.10; 95% CI 0.03, 040). Antenatal care attendance during recent pregnancy was not associated with cessation of breastfeeding at 1 year (URR 1.05; 95% CI 0.87, 1.28).

**Table 3:** Unadjusted and adjusted analysis of factors associated with cessation of breastfeeding at 1 year

Characteristics	Level	Modified Poisson regression analysis			
		uRR	95% CI	aRR	95% CI
Infant sex^a^	Female	Ref		Ref	
	Male	1.35^**^	(1.10,1.66)	1.25^*^	(1.04,1.50)
Maternal age group	≤ 24	Ref		Ref	
	≥ 25	1.21^*^	(1.00,1.46)	1.08	(0.91,1.28)
Maternal parity	1	Ref		Ref	
	2	0.99	(0.76,1.27)	1.02	(0.81,1.30)
	≥ 3	1.23	(0.99,1.53)	1.26^*^	(1.04,1.53)
Attended antenatal care during recent pregnancy	No	Ref			
	Yes	1.05	(0.87,1.28)		
Breastfeeding initiation on same day as birth	No	Ref		Ref	
	Yes	0.10^***^	(0.03,0.40)	0.06^**^	(0.01,0.41)
Time of cotrimoxazole prophylaxis initiation	< 6 weeks	Ref		Ref	
	≥ 6 weeks	0.87^**^	(0.79,0.95)	0.93	(0.86,1.02)

Note: 1) 95% confidence intervals for risk ratios (RR) are in brackets; 2) ^*^ *p* < 0.05, ^**^ *p* < 0.01, ^***^ *p* < 0.001 at 5% level of significance; 3) Adjusted analysis included all statistically significant variable at unadjusted analysis; 4) Ref: Reference category; 5) uRR Unadjusted RR; 6) aRR: Adjusted RR. ^a^The variable infant sex had five missing observations which were not imputed at adjusted analysis

After adjusting for all statistically significant and clinically relevant factors, our results showed that cessation of breastfeeding at 1 year was more likely when the infant was male than female (aRR 1.25; 95% CI 1.04, 1.50) and when the mother was multiparous than primiparous (aRR 1.26; 95% CI 1.04, 1.53). Conversely, cessation of breastfeeding at 1 year was less likely when the infant was initiated on breastfeeding on same-day as birth compared to when the initiation of breastfeeding took place on another day (aRR 0.06; 95% CI 0.01–0.41).

### Qualitative findings

In qualitative data analysis, two major themes emerged regarding cessation of breastfeeding at 1 year: 1) reasons for ceasing breastfeeding at 1 year and this has four sub-themes, and 2) reasons for not ceasing to breastfeed at 1 year, with two sub-themes. These themes and sub-themes are described below.

### Major theme 1: reasons for ceasing to breastfeed at 1 year

#### Sub-theme 1: male infants over breastfeed than females

Mothers living with HIV reported that the male infants breastfeed more frequently than female infants. Accordingly, the male infants were stopped from breastfeeding earlier than the female infants as illustrated in the excerpts below.

*“Those boys can feed, they want to breastfeed every second and I feared my breast may get torn. Personally, I stopped breastfeeding boys at 9 months because feeding them needs too much*” (FGD 1 with mothers living with HIV).

*“They [meaning male infants] feed so much. We [meaning* mothers living with HIV] *don’t get peace at all” (FGD* 2 with mothers living with HIV).

#### Sub-theme 2: maternal literacy and knowledge adequacy about breastfeeding

Participants indicated that the mother’s level of education and knowledge about breastfeeding an infant were important in influencing cessation of breastfeeding at 1 year. Cessation of breastfeeding was mostly observed among educated than non-educated mothers, and among mothers who had knowledge of breastfeeding an infant exposed to HIV compared to those who had no knowledge of breastfeeding.

*“I cannot afford to buy those manufactured milk for my baby because I have to buy food, pay fees and treat the sisters and brothers, so I preferred to breastfeed until the time Musawo (meaning nurse) told me to stop breastfeeding. I was told to stop at 1 year sharp” (FGD 4 with mothers living with HIV).*

*“When mother is educated, she is able to understand what I say, she can even go ahead and read and understand more, she can see the pictures and charts we have here and is able to read compared to those who never went to school, we have to repeat several times before she can understand” (*KII 1 with healthcare provider).

*“Of course, those who are more educated do not disturb us, they follow whatever we say and even and want their babies healthy”* (KII 2 with healthcare provider).

#### Sub-theme 3: support and reminders from the partner

Participants mentioned that for married mothers, the partners played an important role in influencing cessation of breastfeeding through regular reminders on when to stop breastfeeding. In addition, the partners supported them in providing basic family needs. It arose that the partner support and reminders enabled mothers to breastfeed until 1 year.

*“If you are not married you can breast feed your child to whatever time you want or stop whenever you want. But for the married women, your husband will ask you why the baby is not breastfeeding, your husband can also remind you of the recommended time of stopping to breastfeed”* (FGD 3 with mothers living with HIV).

*“A married woman is different from single one, a single mother has no time to breastfeed due to the responsibility of looking for basic needs, but married women get the support from the husband*” (*(*FGD 2 with mothers living with HIV*).*

#### Sub-theme 4: boys can bite the breast once they get teeth

Mothers reported that the male infants bite their breasts frequently than the female infants, especially when they develop teeth. Therefore, male infants were discontinued from breastfeeding much earlier than female infants.

*“The boys, once they get teeth, they can bite so hard and it’s so painful and so I had to stop him from breast feeding” (FGD 1 with mothers living with HIV).*

### Major theme 2: reasons for not ceasing to breastfeed at 1 year

#### Sub-theme 1: insufficient knowledge about breastfeeding

It emerged that mothers who had missed routine health education sessions conducted at the health facility had inadequate knowledge about breastfeeding and this has resulted into not ceasing to breastfeed at 1 year. Second, key informants mentioned that primiparous and secundiparous mothers were less interested in breastfeeding compared to multiparous mothers.

*“When mother is far, she comes late, some times when I have finished health education and she ends up missing. Somehow she may fail to follow the guideline and even breastfeed more than one year or believe that not breastfeeding when the teeth are out is the better” (KII 3 with healthcare provider).*

*“The girls who have one or two children do not want to breastfeed, they say that they do not want their breasts to fall*” (KII 1 with healthcare provider).

#### Sub-theme 2: girls feed a bit less

Mothers were concerned about the feeding habits of male versus female infants. Many of the mothers reported that the female infants breastfeed comparatively less than the male infants. Consequently, female infants were not discontinued from breastfeeding before 1 year compared to male infants.

*“They [meaning male infants] feed so much. We [meaning* mothers living with HIV] *don’t get peace at all, the girls feed a bit less and we can manage our daily activates while breastfeeding” (FGD* 2 with mothers living with HIV).

*“The girls feed a bit less and we can manage our daily activates while breastfeeding”* (FGD 2 with mothers living with HIV).

## Discussion

We studied cessation of breastfeeding at 1 year among infants born to mothers living with HIV in a large peri-urban health facility in Wakiso district, Uganda. Our data shows that 64% of mothers living with HIV cease breastfeeding at 1 year which is distant from the Uganda National EMTCT policy recommended target of 100% [20]. This suggests that a considerable proportion of infants (36%) are not ceased or discontinued from breastfeeding at 1 year as recommended [8, 9]. These findings have important implications regarding the risk of infant mortality and HIV acquisition. First, cessation of breastfeeding before 1 year (suboptimal infant feeding) increases the risk of malnutrition [8, 9, 21]. Second, breastfeeding for a longer duration than 1 year places the infants at increased risk of HIV acquisition [8, 9, 22]. In general, both breastfeeding practices increase the risk of mortality among infants exposed to HIV. Our findings therefore indicate that context-specific interventions are needed to improve cessation of breastfeeding at 1 year in order to circumvent the negative consequences of untimely (earlier or later than 1 year) cessation of breastfeeding, and to enable the health facility achieve the EMTCT goal of less than 5% HIV transmission in a breastfeeding population [20]. Previous studies in Ethiopia report 34% [23] and 45.5% [24] cessation of breastfeeding at 1 year, which is relatively lower that what we report in this study. The observed differences could possibly be attributed to cultural variations in breastfeeding between the two countries. In Uganda, breastfeeding is a norm and is embraced by almost all cultures, except for medical or public health measures such as PMTCT.

Our study shows that cessation of breastfeeding at 1 year is more likely for male than female infants. This finding is surprising as one would not expect any differences in breastfeeding duration with respect to sex. We did not find biologically plausible reasons to explain the association between sex and cessation of breastfeeding from published studies. Qualitative results show that female infants breastfeed far less than male infants.

The mothers mentioned that breastfeeding male infants has a draining and exhausting effect compared to female infants. Accordingly, the male infants are stopped from breastfeeding earlier than 1 year compared to the female infants. Despite these differences, our finding seems to suggest that routine provision of health education on the benefits of infant breastfeeding until 1 year and disadvantages of ceasing to breastfeed earlier than 1 year among mothers living with HIV remains important in EMTCT.

We found multiparous mothers living with HIV are more likely to cease breastfeeding at 1 year relative to primiparous mothers. This might have resulted from differences in experience with the EMTCT program, with multiparous mothers having sufficient knowledge and experience compared to primiparous mothers. Our finding is consistent with that of Hackman et al. (2015) [24] who observed that maternal parity determines the time at which breastfeeding ceases. Hackman et al. (2015) [24] found that multiparous mothers living with HIV had significantly longer breastfeeding duration than primiparous mothers. One of the reasons attributed to this finding is knowledge inadequacy among primiparous mothers about the importance of breastfeeding since majority were unwilling to breastfeed due to fear of developing sagging breasts. Consistent with our results, a previous study in Ethiopia shows that mothers who are deficient in EMTCT knowledge are less likely to adhere to breastfeeding guidelines [25]. Our finding could also imply that experience with the EMTCT program is a crucial factor for cessation of breastfeeding given the differences between primiparous and multiparous mothers. In general, our result is an indication that primiparous mothers living with HIV might benefit from targeted health education messages compared to multiparous mothers.

Our study shows that initiation of breastfeeding on same-day as birth reduces the likelihood of cessation of breastfeeding at 1 year compared to initiation of breastfeeding on another day.

Failure to initiate breastfeeding on same-day as birth could be explained by several reasons namely, insufficiency of breast milk, low birthweight, and premature birth among others [26].

We hypothesized that mothers who initiate breastfeeding on same-day as birth are those knowledgeable about the importance of breastfeeding in the first few hours of birth. Our finding implies that healthcare providers should emphasize the importance of initiating breastfeeding within the first hour of birth to all mothers living with HIV. However, cessation of breastfeeding at 1 year should be strongly emphasized to prevent mother to child transmission of HIV. Additionally, further research is needed to understand barriers to uptake of Option B policy on cessation of breastfeeding at 1 year in this setting and similar areas.

### Study strengths and limitations

This study has several strengths. First, it is among the first few studies in Uganda to examine the cessation of breastfeeding at 1 year among mothers living with HIV per the national HIV prevention and treatment guideline. Second, the use of qualitative data to enrich and triangulate the quantitative findings is another strength. However, a number of limitations should be considered in the interpretation of the results. We used a retrospective cohort study design and this design by default does not demonstrate causation rather association. Our study was conducted in a peri-urban health facility so the results might not be generalizable to rural health facilities. We did not study several potential confounders such as healthcare workers knowledge about EMTCT and attitude, the role of mentor mothers, HIV stigma, maternal decision-making power, family support system, and antenatal care attendance because we used secondary data and this was an important limiting factor. However, we tried to overcome this problem by incorporating qualitative data to enrich the quantitative data although peer mothers who held rich experience about breastfeeding were not interviewed. Although we encouraged equal participation during the FGD, it is likely that the inclusion of mothers of all ages in the same group might have prevented younger mothers from fully expressing themselves.

There is also the possibility that data recorded in the registers might have recording and transcription errors, although we attempted to verify the data for accuracy. Our study could not conclude on the outcomes of infants transferred to other health facilities and those lost to follow-up because it was logistically impractical to obtain such data. The non-sequencing of qualitative and quantitative data collection methods limited us from exploring the reasons as to why the female infants are not ceased from breastfeeding at 1 year compared to the male infants. Lastly, our sample size was relatively small despite the inclusion of all mother-baby pairs in the cohort.

## Conclusions

Our study shows that 64% of mothers living with HIV cease breastfeeding at 1 year, which is substantially lower than the recommended national target of 100% under the EMTCT policy. Healthcare systems should therefore strengthen the implementation and adoption of the EMTCT policy. Our data shows that cessation of breastfeeding at 1 year is more likely for male than female infants, among multiparous than primiparous mothers living with HIV, and less likely when initiation of breastfeeding occurred on same-day as birth relative to another day. The qualitative findings showed that four sub-themes namely male infants over breastfeed than females, maternal literacy and knowledge adequacy about breastfeeding, support and reminders from the partner, and boys can bite once they get teeth emerged as reasons for cessation of breastfeeding at 1 year. Conversely, two sub-themes namely insufficient knowledge about breastfeeding and girls feed a bit less emerged as reasons for not ceasing to breastfeed at 1 year. We recommend the strengthening of health education messages on infant feeding in the context of HIV among mothers/expectant women living with HIV. Further robust studies are needed to underscore reasons for preferential breastfeeding of female relative to male infants.

## Data Availability

The datasets used and/or analyzed during the current study are available from the corresponding author on reasonable request.

## Abbreviations

aRR: Adjusted Risk Ratio
EID: Early infant diagnosis of HIV
EMTCT: Elimination of mother to child transmission of HIV
HIV: Human Immunodeficiency Virus
uRR: Unadjusted Risk Ratio
WHO: World Health Organization
